# Effect of immobilized cells in calcium alginate beads in alcoholic fermentation

**DOI:** 10.1186/2191-0855-3-31

**Published:** 2013-05-30

**Authors:** Juliana C Duarte, J Augusto R Rodrigues, Paulo J S Moran, Gustavo P Valença, José R Nunhez

**Affiliations:** 1 Institute of Chemistry, University of Campinas, Campinas, SP 13083-970, Brazil; 2 School of Chemical Engineering, University of Campinas, Campinas, SP 13083-852, Brazil

**Keywords:** Immobilized cells, Fermentation, Ethanol, Glucose, Sucrose, Alginate

## Abstract

*Saccharomyces cerevisiae* cells were immobilized in calcium alginate and chitosan-covered calcium alginate beads and studied in the fermentation of glucose and sucrose for ethanol production. The batch fermentations were carried out in an orbital shaker and assessed by monitoring the concentration of substrate and product with HPLC. Cell immobilization in calcium alginate beads and chitosan-covered calcium alginate beads allowed reuse of the beads in eight sequential fermentation cycles of 10 h each. The final concentration of ethanol using free cells was 40 g L^-1^ and the yields using glucose and sucrose as carbon sources were 78% and 74.3%, respectively. For immobilized cells in calcium alginate beads, the final ethanol concentration from glucose was 32.9 ± 1.7 g L^-1^ with a 64.5 ± 3.4% yield, while the final ethanol concentration from sucrose was 33.5 ± 4.6 g L^-1^ with a 64.5 ± 8.6% yield. For immobilized cells in chitosan-covered calcium alginate beads, the ethanol concentration from glucose was 30.7 ± 1.4 g L^-1^ with a 61.1 ± 2.8% yield, while the final ethanol concentration from sucrose was 31.8 ± 6.9 g L^-1^ with a 62.1 ± 12.8% yield. The immobilized cells allowed eight 10 h sequential reuse cycles to be carried out with stable final ethanol concentrations. In addition, there was no need to use antibiotics and no contamination was observed. After the eighth cycle, there was a significant rupture of the beads making them inappropriate for reuse.

## Introduction

Due to the gradual depletion of crude oil and environmental problems caused as a result of the growing consumption of oil and its derivatives, biomass and bioenergy have recently been attracting more attention. Therefore, it is important to study and develop alternatives that are both renewable and environmentally friendly. Ethanol has been considered an alternative to oil, since it is more environmentally friendly and is a renewable energy source (Bai et al. 2008; Lin and Tanaka 2006; Liu et al. 2009; Najafpour et al. 2004; Rattananpan et al. 2011; Xu et al. 2005). In Brazil ethanol is produced from sugar cane (Andrade 2007; Borges 2008; Pacheco 2010; Wendhausen et al. 2001) and the country is the largest exporter and second largest producer of ethanol, second only to the U.S., where it is produced primarily from corn (Borges 2008; Pacheco 2010). In addition, Brazil has favorable soil and climatic conditions and is recognized as a technology leader in ethanol production from sugar cane with the world’s greatest potential for increasing its production of biofuels (Borges 2008; Pacheco 2010; Rodrigues 2011). In Brazil the development of technologies that can improve the performance of ethanol production has gained considerable importance over the past 35 years. In this scenario, cell immobilization has been used for ethanol production in bioreactors in order to decrease the inhibition caused by high concentrations of substrate and product, thus increasing ethanol production and reducing its costs. There are several studies reporting the use of immobilized cells in various supports for a more economical production of ethanol. These systems are attractive and promising, since production is higher than that observed for free cells (Bai et al. 2008; Lin and Tanaka 2006; Najafpour et al. 2004; Inloes et al. 1983; Puligundla et al. 2011; Yao et al. 2011; Ylitervo et al. 2011; Yu et al. 2010). Each immobilization method has its advantages and disadvantages. The procedure of immobilization in alginate beads is not only inexpensive but also easy to carry out and provides extremely mild conditions, so there is a higher potential for industrial application (Zhou et al. 2010). Calcium alginate beads are one of the most commonly used supports for the immobilization of cells. They offer several advantages as a support, such as good biocompatibility, low cost, easy availability, and ease of preparation. However, there are some disadvantages associated with their use, such as gel degradation, severe mass transfer limitations, low mechanical strength (causing cells to be released from the support), and large pore size (Zhou et al. 2010; Bangrak et al. 2011). In order to optimize encapsulation efficiency and avoid the release of cells from the support, various methods have been proposed (Roca et al. 1996; Winkelhausen et al. 2010), including covalent cross-linking with polymers such as chitosan and glutaraldehyde (Zhou et al. 2010).

Immobilized cells have some advantages over free cells, such as higher cell density per volume of reactor, easier separation from the reaction medium, continuous operation without cells being carried away downstream, reduction of the adaptation phase (lag phase), a higher substrate conversion, less inhibition by products, reduced reaction time, and control of cell replication (biomass growth) (Wendhausen 1998).

This work studies the immobilization of cells of the JAY 270 strain of the yeast *Saccharomyces cerevisiae,* using calcium alginate and chitosan-covered calcium alginate beads. Glucose and sucrose are used as carbon sources for the fermentation. Chitosan, a linear polysaccharide composed of randomly β-(1-4)-linked-D-glucosamine (deacetylated unit) and N-acetyl-D-glucosamine (acetylated unit) (Canella and Garcia 2001), was used as the external layer of the calcium alginate beads. Alginate and chitosan, which are polysaccharide biopolymers, have been the focal point of an expanding number of studies that address their potential for use in enzyme/cell encapsulation (Zhou et al. 2010). When calcium alginate is mixed with chitosan, a strong ionic interaction occurs between the amino groups of chitosan and carboxyl groups of alginate for the formation of a polyelectrolyte complex (PEC), which results in better mechanical properties of the support (Ngah and Fatinathan 2008; Rodrigues 2008; Shu and Zhu 2002; Xu et al. 2007).

## Materials and methods

### Materials

#### Microorganism

The JAY 270 strain of *Saccharomyces cerevisiae* was obtained from the Institute of Biology at UNICAMP. This strain had been studied in previous experiments (Argueso et al. 2009) and showed a good ethanol production and high biomass production.

#### Chemicals

Glucose and acetic acid were obtained from Synth; sucrose, from Aldrich; yeast extract, from Oxoid; peptone and malt extract, from Himedia, sodium alginate (average viscosity) and chitosan (medium molecular weight, degree of deacetylation 75-85%), from Sigma-Aldrich; and calcium chloride, from Aldrich Chemical Company, Inc.

### Methods

#### Growing medium

The yeast cells were grown in a sterile solution (121°C, 15 min.) containing 10 g L^-1^ glucose, 5 g L^-1^ peptone, 3 g L^-1^ yeast extract, and 3 g L^-1^ malt extract. After one day, the mixture was centrifuged (2000 rpm, 20 min.) and suspended in sterile water (0.10 L).

#### Fermentation medium with glucose

A sterile solution (121°C, 20 min.) was prepared with 100 g L^-1^ glucose, 20 g L^-1^ peptone, and 10 g L^-1^ yeast extract. This solution was used in the batch fermentation experiments.

#### Fermentation medium with sucrose

A sterile solution (121°C, 20 min.) was prepared with 100 g L^-1^ sucrose, 20 g L^-1^ peptone, and 10 g L^-1^ yeast extract. This solution was also used in the batch fermentation experiments.

#### Calcium alginate beads

For the immobilization in beads, 3% (w/v) sodium alginate was dissolved in 0.10 L water and added to a 0.10 L suspension of *S. cerevisiae* in a beaker. The solution was mildly shaken. A CaCl_2_ solution with a final concentration of 2% (w/v) was prepared in a separate beaker. The mixture containing the cells and the sodium alginate was added dropwise to 0.150 L of the CaCl_2_ solution using a 0.010 L syringe. The beads were hardened in this solution for 1 h. After hardening in the CaCl_2_ solution, the beads were rinsed with sterile water to be used thereafter in the fermentation experiments. The beads were got with a diameter approximately between 3 to 4 mm.

#### Chitosan-covered calcium alginate beads

For the immobilization in beads, 3% (w/v) sodium alginate was dissolved in 0.10 L water and added to a 0.10 L suspension of *S. cerevisiae* in a beaker. The solution was mildly shaken. A CaCl_2_ solution with a final concentration of 2% (w/v) was prepared in a separate beaker. A solution of 0.25% (w/v) chitosan in 5% acetic acid was prepared in another beaker. The mixture containing the cells and the sodium alginate was added dropwise to 0.150 L of the CaCl_2_ solution using a 0.010 L syringe. The beads were hardened in this solution for 1 h. After hardening in the CaCl_2_ solution, the beads were rinsed with sterile water and then added to the 0.25% chitosan solution and kept under mild shaking for 30 minutes. The beads were then rinsed with sterile water to be used thereafter in the fermentation experiments. The beads were got with a diameter approximately between 3 to 4 mm.

#### Batch fermentation experiments using the immobilized cells

Approximately 100 g of calcium alginate beads was added to a 1.0 L Erlenmeyer flask containing 0.20 L of the fermentation medium with glucose or sucrose as carbon source. All steps prior to fermentation were carried out under sterile conditions. The procedure described above was carried out for chitosan-covered calcium alginate beads. The four flasks were sealed with a cotton plug and placed in an orbital shaker for 10 h at 30°C and 200 rpm. Samples were collected during the fermentation period. After 10 h, the beads were filtered and rinsed with sterile water and added to a fresh fermentation medium (with glucose or sucrose as carbon sources for calcium alginate and chitosan-covered calcium alginate beads) as described above. This procedure was repeated eight times for the successive fermentation cycles.

#### Batch fermentation experiments using free cells

In order to compare the performance of the immobilized cells, a parallel experiment using free cells for the fermentation was carried out using the same mass of cells as that used in the immobilized beads. The experiments were carried out under the same experimental conditions with either glucose or sucrose as carbon source.

#### Analytical methods

Ethanol, glucose, and sucrose concentrations were determined using high-performance liquid chromatography equipment (Agilent Technologies 1200 series) equipped with a Aminex ® HPX-87H column (Biorad) and refractive index detector (RID). Cell growth was determined by optical density at 600 nm in an Agilent HP 8453 spectrophotometer using the fermentation medium as blank.

## Results

**Figure 1:**
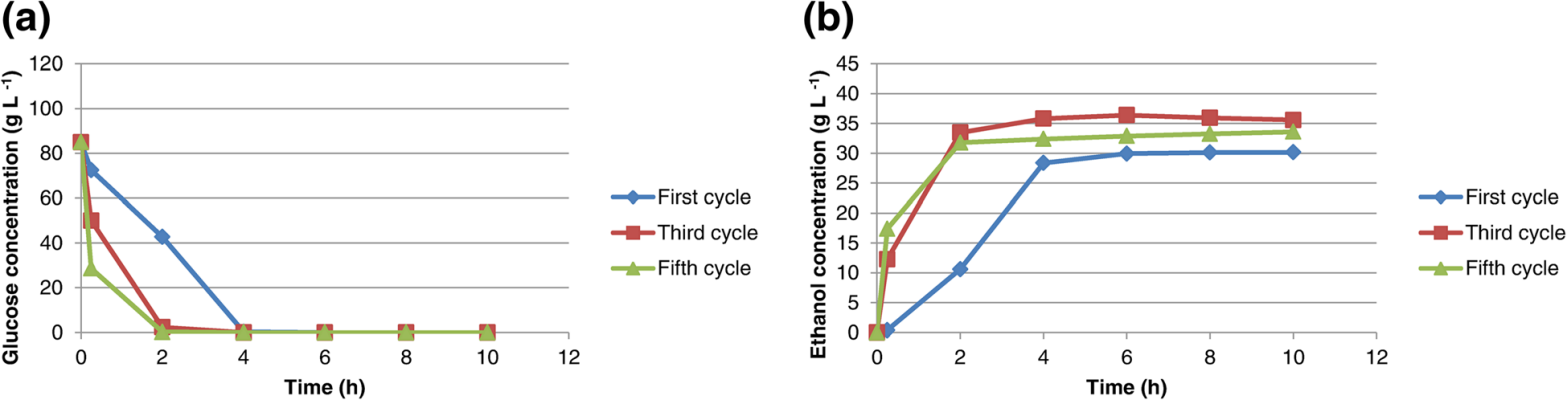
**Profiles of first, third, and fifth reuse cycles of immobilized cells of *Saccharomyces cerevisiae *in CAB during fermentations of glucose.** (**a**) glucose consumption profile for immobilized cells in calcium alginate beads during first, third, and fifth cycle (10 h each cycle); (**b**) ethanol concentration profile for immobilized cells in calcium alginate beads during first, third, and fifth cycle (10 h each cycle).

**Figure 2:**
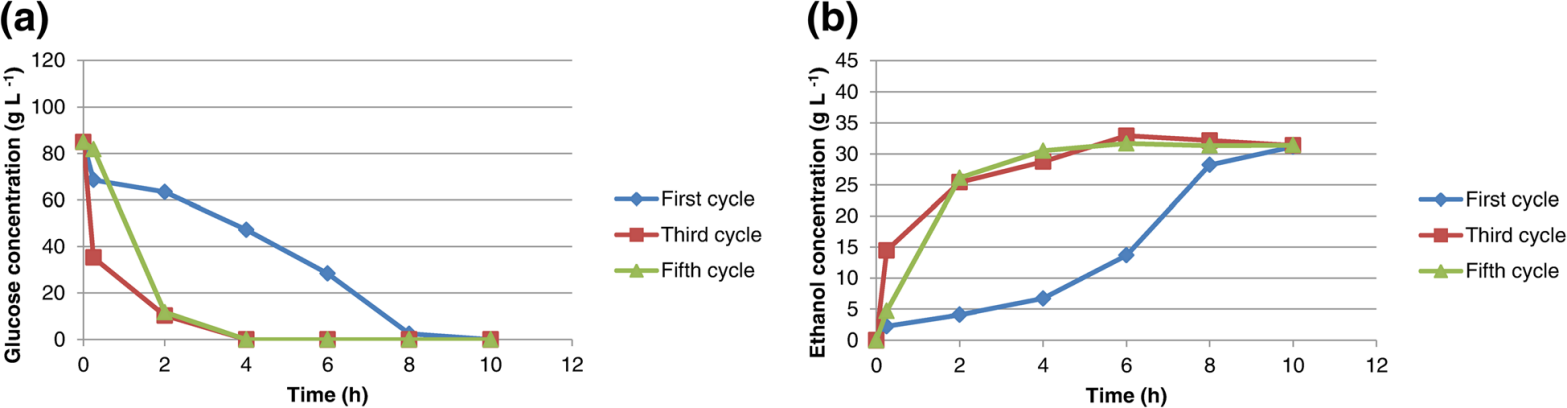
**Profiles of first, third, and fifth reuse cycles of immobilized cells of *Saccharomyces cerevisiae *in CCCAB during fermentations of glucose.** (**a**) glucose consumption profile for immobilized cells in chitosan-covered calcium alginate beads during first, third, and fifth cycle (10 h each cycle); (**b**) ethanol concentration profile for immobilized cells in chitosan-covered calcium alginate beads during first, third, and fifth cycle (10 h each cycle).

**Figure 3:**
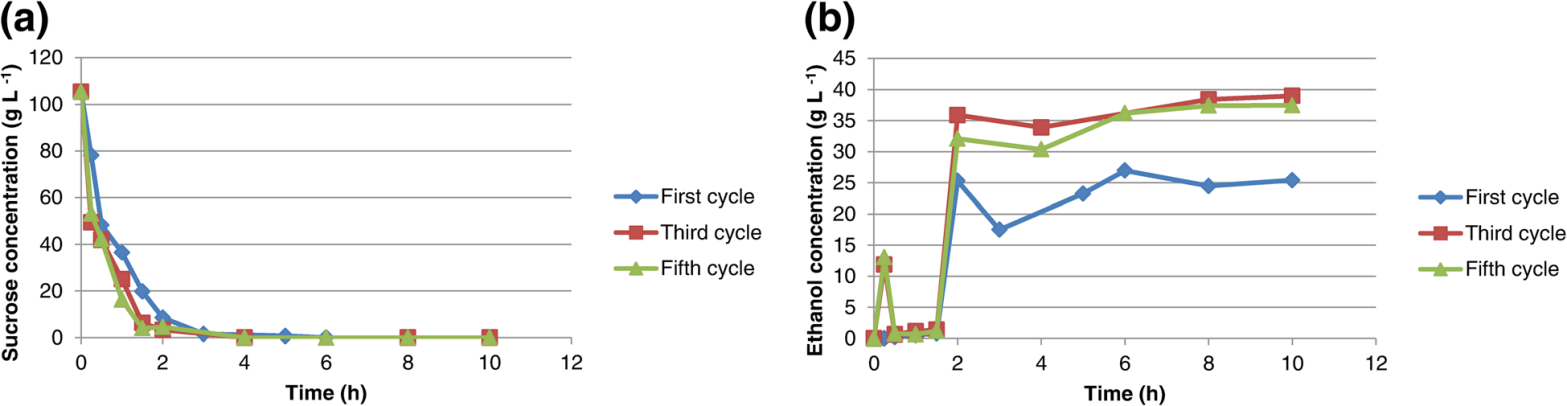
**Profiles of first, third, and fifth reuse cycles of immobilized cells of *Saccharomyces cerevisiae *in CAB during fermentations of sucrose.** (**a**) sucrose consumption profile for immobilized cells in calcium alginate beads during first, third, and fifth cycle (10 h each cycle); (**b**) ethanol concentration profile for immobilized cells in calcium alginate beads during first, third, and fifth cycle (10 h each cycle).

**Figure 4:**
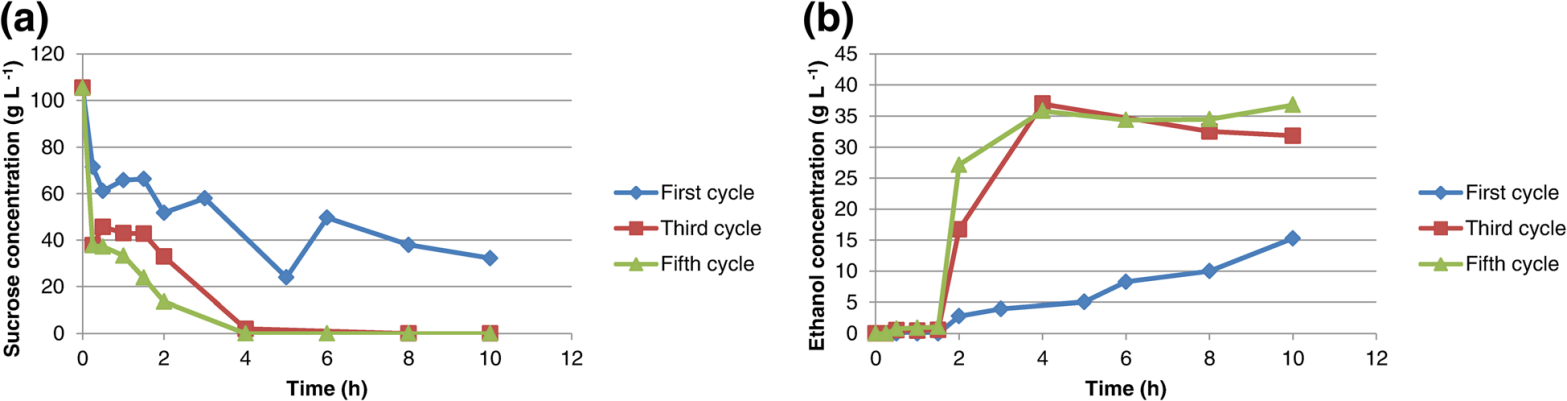
**Profiles of first, third, and fifth reuse cycles of immobilized cells of *Saccharomyces cerevisiae *in CCCAB during fermentations of sucrose.** (**a**) sucrose consumption profile for immobilized cells in chitosan-covered calcium alginate beads during first, third, and fifth cycle (10 h each cycle); (**b**) ethanol concentration profile for immobilized cells in chitosan-covered calcium alginate beads during first, third, and fifth cycle (10 h each cycle).

**Figure 5:**
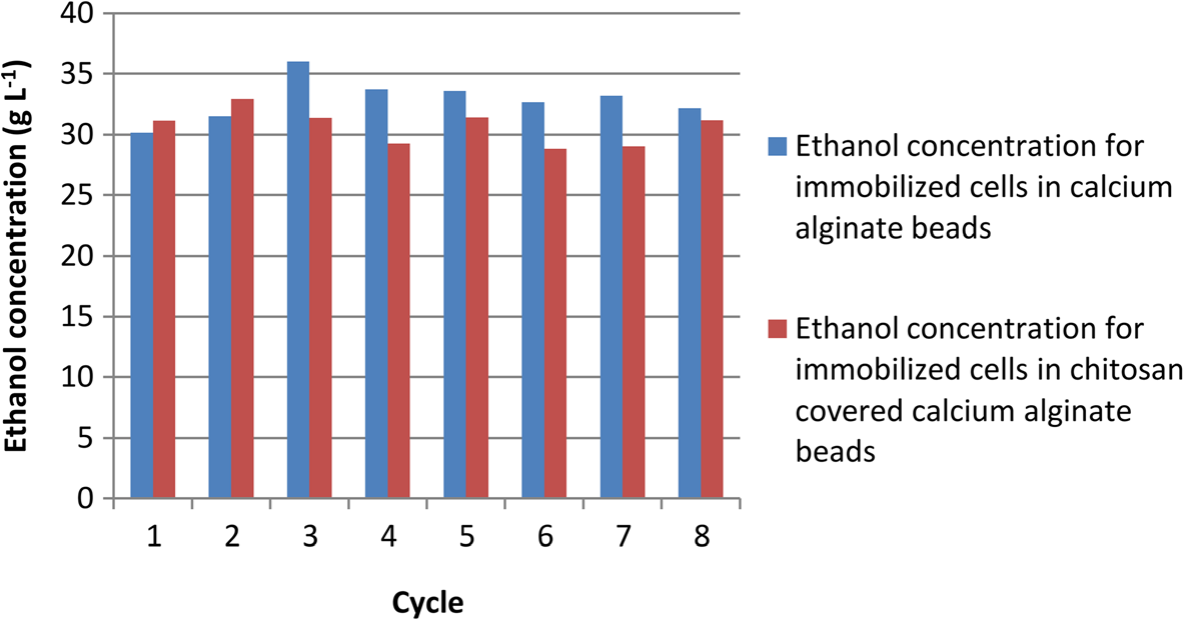
Final ethanol concentration at the end of each cycle using glucose as carbon source.

**Figure 6:**
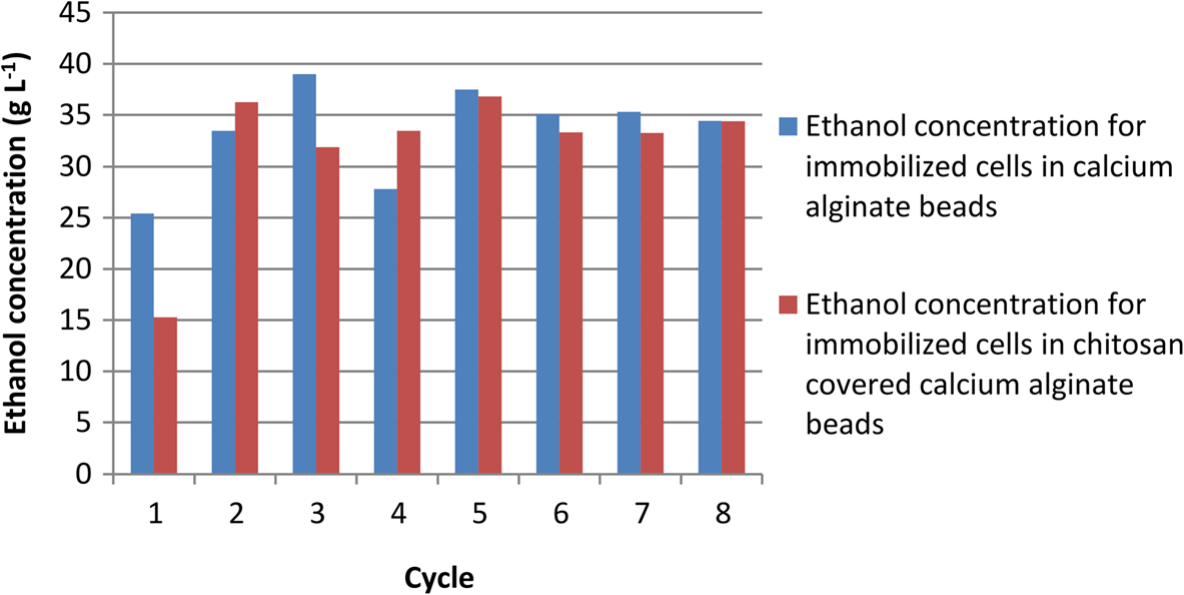
Final ethanol concentration at the end of each cycle using sucrose as carbon source.

Eight sequential fermentation cycles using immobilized cells in calcium alginate and chitosan-covered calcium alginate beads were carried out with 100 g L^-1^ glucose or sucrose as carbon source. The cycles lasted 10 h each and every experiment used approximately 6 g of wet yeast cells. When mixed with the alginate, these cells had a final weight of 100 g, so the immobilized cell concentration in the beads was approximately 60 g L^-1^. Ethanol yield and cell growth in aqueous medium are slightly different in fermentation systems for free cells from those for immobilized cells. The ethanol concentration, yield, and cell growth in aqueous medium for glucose or sucrose fermentation are shown in Tables 1 and 2, respectively. The yield is defined as the amount of ethanol produced in relation to the maximum ethanol that could be produced from the carbon source (glucose or sucrose). For example, for glucose, 1 mole of glucose could produce 2 moles of ethanol if the conversion were 100%. For free cells, the yield is the value after one experiment; however, since immobilized cells were used in eight cycles, the yield is calculated as the average of the yields for all eight cycles. The same applies to cell growth in aqueous medium and ethanol concentration. As shown in Table 1, the yields from glucose for free cells, immobilized cells in calcium alginate beads, and immobilized cells in chitosan-covered calcium alginate beads are 78%, 64.5 ± 3.4%, and 61.1 ± 2.8%, respectively. As shown in Table 2, the yields from sucrose for free cells, immobilized cells in calcium alginate beads, and immobilized cells in chitosan-covered calcium alginate beads are 74.3%, 64.5 ± 8.6%, and 62.1 ± 12.8%, respectively. A low yield has been observed in fermentations with immobilized cells, which might be due to diffusional problems because entrapped cells may not have effective contact with essential nutrients in the broth or the cells may be inhibited by the product (Nikolic et al. 2010). In this article, cell growth in an aqueous medium is defined as the number of cells formed during fermentation. For the free cells, cell growth in aqueous medium is estimated as the total number of cells after the cycle less the cells introduced at the beginning of fermentation. For immobilized cells, cell growth in aqueous medium was estimated as the number of cells released from the beads. It is possible that some cell growth might have occurred inside the beads. However, since all beads were used for eight cycles, cell growth was not determined. The fermentations using calcium alginate and chitosan-covered calcium alginate beads with glucose as carbon source are shown in Figures 1 and 2, respectively. The curves are for the first, third, and fifth cycles. The maximum ethanol concentration was reached after 4 h of reaction for the immobilized cells in beads without chitosan and after 6 h for those immobilized in beads with chitosan. The fermentations using calcium alginate and chitosan-covered calcium alginate beads with sucrose as carbon source are shown in Figures 3 and 4, respectively. These figures contain the curves for the first, third, and fifth cycles. Unlike the fermentations using glucose, the maximum ethanol concentration was reached after 2 h of reaction for the immobilized cells without chitosan and after 4 h for those with chitosan. It can be observed in all the figures that the increase in ethanol concentration and the decrease in sugar concentration in the first cycle are considerably lower than those observed in the third and fifth cycles. One possible explanation is that during the first cycle, cells are in an adaptation phase and they might be suffering stress caused by immobilization. In addition, cell growth within the beads should contribute to the increased final ethanol concentration in later cycles. The only data on ethanol concentration for the second, fourth, sixth, seventh, and eighth cycles is the concentration at the beginning and at the end of the experiment. The final ethanol concentrations at the end of each cycle are shown in Figures 5 and 6 for glucose and sucrose, respectively. It can be observed that the final ethanol concentration was considerably constant after the first cycle in both figures when using immobilized cells in calcium alginate beads and in chitosan-covered calcium alginate beads.

**Table 1:** **Ethanol concentration, yield and cell growth in aqueous medium after glucose fermentation by free cells and immobilized cells of *Saccharomyces cerevisiae***

	**Free cells**	**Immobilized cells CAB**^**a**^	**Immobilized cells CCCAB**^**b**^
**Ethanol concentration (g L**^**-1**^**)**	40	32.9 ± 1.7^*^	30.7 ± 1.4^*^
**Yield (%)**	78	64.5 ± 3.4^*^	61.1 ± 2.8^*^
**Cell growth in aqueous medium (g L**^**-1**^**)**	17.6	0.19 ± 0.12^*^	0.09 ± 0.05^*^

^a^ CAB: calcium alginate beads.

^b^ CCCAB: chitosan-covered calcium alginate beads.

^*^ Average data for eight successive fermentation cycles.

**Table 2:** **Ethanol concentration, yield and cell growth in aqueous medium after sucrose fermentation by free cells and immobilized cells of*Saccharomyces cerevisiae***

	**Free cells**	**Immobilized cells CAB**^**a**^	**Immobilized cells CCCAB**^**b**^
**Ethanol concentration (g L**^**-1**^**)**	40	33.5 ± 4.6^*^	31.8 ± 6.9^*^
**Yield (%)**	74.3	64.5 ± 8.6^*^	62.1 ± 12.8^*^
**Cell growth in aqueous medium (g L**^**-1**^**)**	15.6	0.40 ± 0.38^*^	0.26 ± 0.30^*^

^a^ CAB: calcium alginate beads.

^b^ CCCAB: chitosan-covered calcium alginate beads.

^*^ Average data for eight successive fermentation cycles.

## Discussion

Cell immobilization in beads offers important advantages, such as ease of cell separation from the medium, a decrease in costs due to cell reuse in subsequent reaction cycles, and a reduced possibility of contamination. Furthermore, the results of cell immobilization showed a stable final ethanol concentration throughout the cycles (Figures 5 and 6) so these immobilized cells could be employed in a continuous process in a tubular reactor. There was no need to use antibiotics during fermentation and no contamination was observed throughout the experiment. Contamination is a serious technical and economic problem with the use of free cells in mills where cells must be recycled.

**Figure 7:**
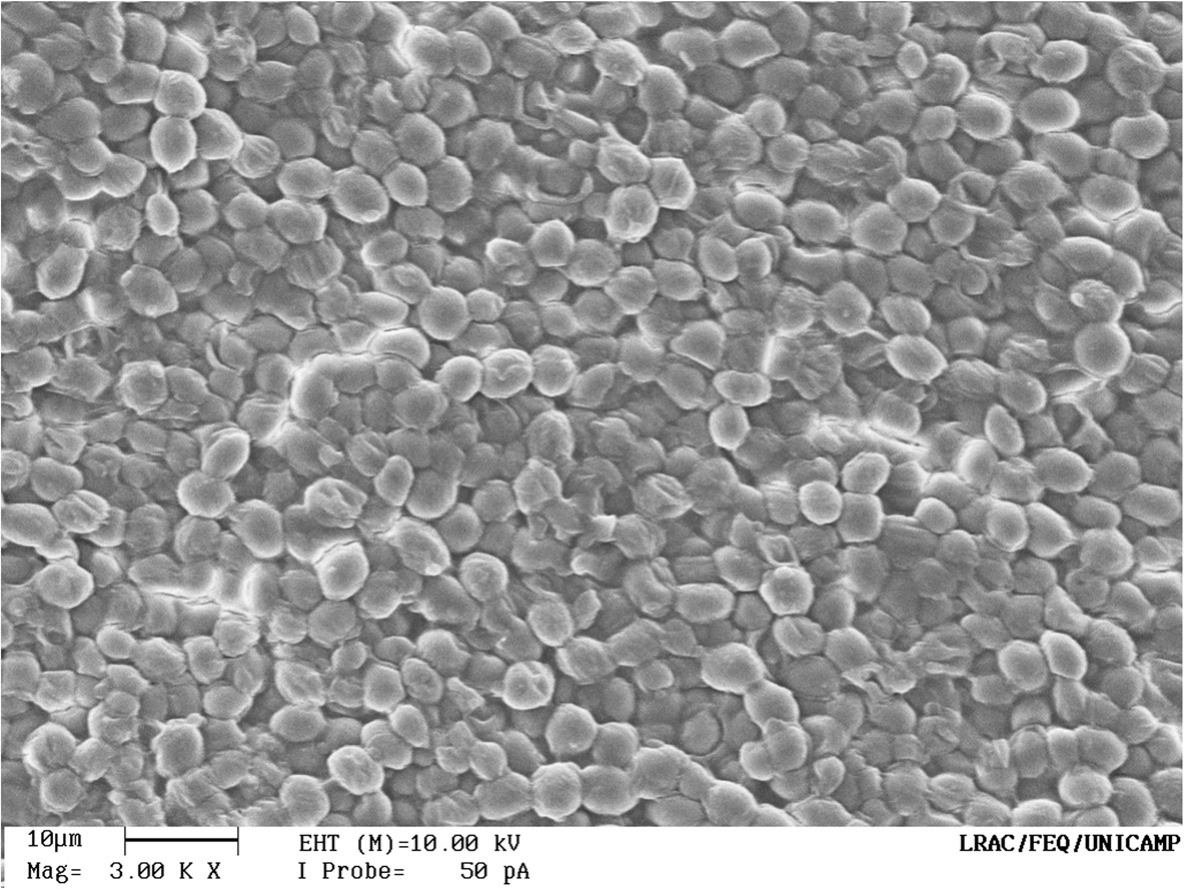
SEM image to the calcium alginate bead.

**Figure 8:**
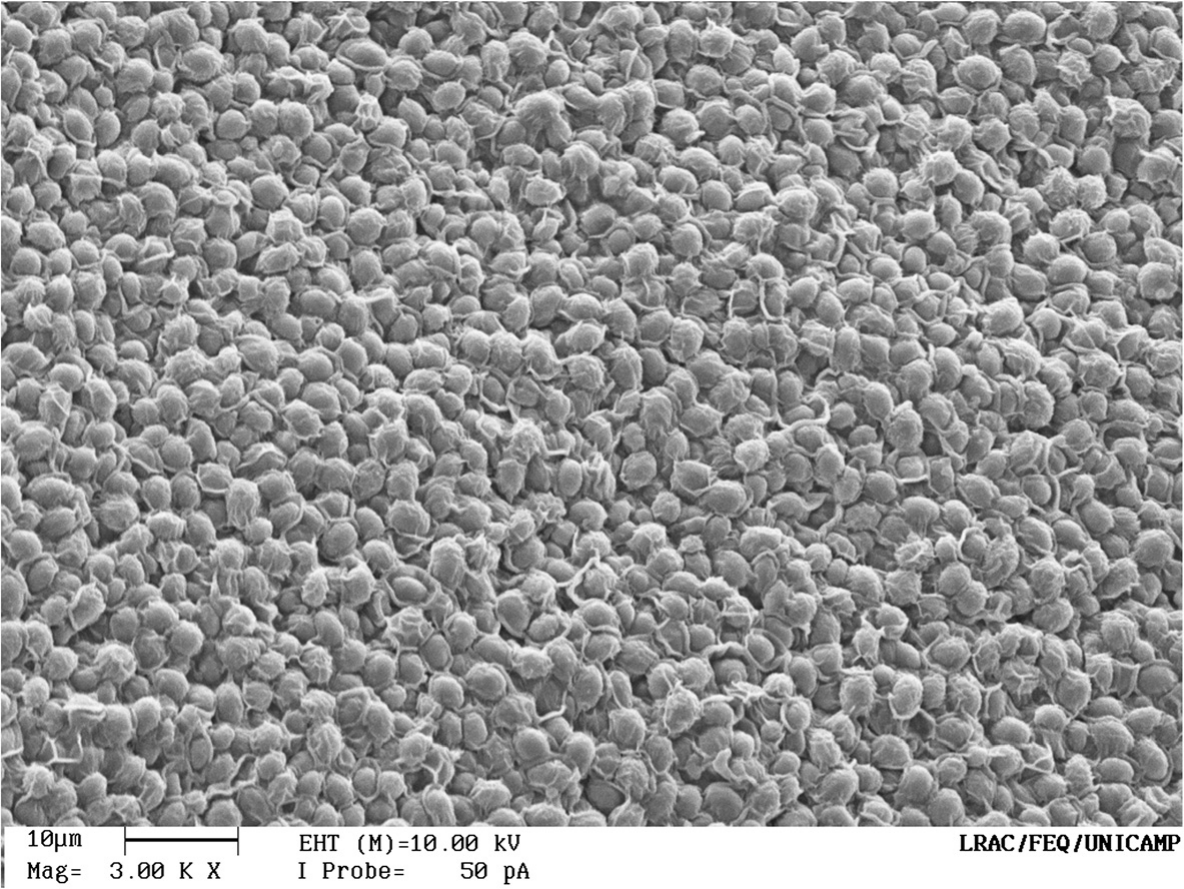
SEM image to the chitosan covered calcium alginate bead.

The final ethanol concentration and yield were higher for free cell fermentation systems using glucose or sucrose than for systems carried out with immobilized cells. Chitosan-covered calcium alginate beads were not mechanically more resistant than beads without chitosan. When using chitosan, cell growth in aqueous medium was slightly lower than that for the beads without it. This had been expected, since chitosan acts as a barrier to cell release. Figures 7 and 8 show the scanning electron microscope (SEM) images to the calcium alginate beads and to the chitosan-covered calcium alginate beads, respectively. It can be noticed in Figure 8, a sort of chitosan film cover the yeast cells, keeping them more adherent to the bead surface. These SEM images of the beads were acquired before to use them in fermentations. The final ethanol concentration using immobilized cells was similar, even after eight sequential fermentation cycles. For free cells, the final ethanol concentration was 40 g L^-1^, but this experiment was carried out without cell reuse. For immobilized cells, it was about 30 g L^-1^ in each cycle with cell reuse. Our results show an improvement over those observed by Ghorbani et al. (2011), who showed a final ethanol concentration of 19.15 g L^-1^ using sodium-alginate immobilized yeast in an immobilized cell reactor (ICR) with a feed containing 150 g L^-1^ of molasses. Using a 10% initial glucose concentration Birol et al. (1998) observed a final ethanol concentration of 41 g L^-1^, which was similar to the one we obtained for free cell fermentation. Our results for immobilized cells are also similar to those reported by Shafaghat et al. (2011), where continuous ethanol fermentation in ICR using molasses and immobilized cells in calcium alginate achieved a maximum ethanol concentration of 45.5 g L^-1^ in an experiment with an initial sugar concentration of 100 g L^-1^. It was observed (Eiadpum et al. 2012) that a co-culture of *Kluyveromyces marxianus* and *Saccharomyces cerevisiae* immobilized on thin-shell silk cocoon was effective for ethanol fermentation at a high-temperature, generating ethanol concentrations of 81.4 and 77.3 g L^-1^ when using temperatures of 37 and 40°C and an initial sugar concentration of 220 g L^-1^. Our results show that ethanol conversion time was longer on chitosan-covered calcium alginate beads than on calcium alginate beads. It was noticed that the chitosan-covered calcium alginate beads were slightly more robust than the calcium alginate beads after eight sequential fermentation cycles. However, this difference was not significant. Final ethanol concentration is due mostly to the immobilized cells since the cell concentration in aqueous medium due to the release from beads is negligible when compared to the cell concentration in the beads. It was possible to perform eight sequential cycles of 10 h each using immobilized cells. Since the maximum ethanol concentration was reached in 6 h for the chitosan-covered beads, it may be possible to increase significantly the number of cycles if the cycle time is reduced to 6 h.

In conclusion, with immobilized cells it was possible to carry out eight sequential reuse cycles, generating a stable final ethanol concentration in each cycle. Therefore, this study suggests that the beads' robustness allows potential application in a continuous process in a tubular reactor. In addition, there was no need to use antibiotics and contamination was not observed. In comparing calcium alginate beads and chitosan-covered calcium alginate beads, it was observed that the chitosan cover did not improve the beads' robustness and, moreover, substrate consumption and ethanol production were slower because chitosan acted as a barrier to substrates and products.

## Competing interests

The authors declare that they have no competing interests.
